# Linc-ing Circulating Long Non-coding RNAs to the Diagnosis and Malignant Prediction of Intraductal Papillary Mucinous Neoplasms of the Pancreas

**DOI:** 10.1038/s41598-017-09754-5

**Published:** 2017-09-05

**Authors:** Jennifer B. Permuth, Dung-Tsa Chen, Sean J. Yoder, Jiannong Li, Andrew T. Smith, Jung W. Choi, Jongphil Kim, Yoganand Balagurunathan, Kun Jiang, Domenico Coppola, Barbara A. Centeno, Jason Klapman, Pam Hodul, Florian A. Karreth, Jose G. Trevino, Nipun Merchant, Anthony Magliocco, Mokenge P. Malafa, Robert Gillies

**Affiliations:** 1 Departments of Cancer Epidemiology, Moffitt Cancer Center and Research Institute, Tampa, Florida USA; 2 Gastrointestinal Oncology, Moffitt Cancer Center and Research Institute, Tampa, Florida USA; 3 Biostatistics and Bioinformatics, Moffitt Cancer Center and Research Institute, Tampa, Florida USA; 4 Molecular Genomics Core Facility, Moffitt Cancer Center and Research Institute, Tampa, Florida USA; 5 Diagnostic Imaging and Interventional Radiology, Moffitt Cancer Center and Research Institute, Tampa, Florida USA; 6 Cancer Imaging and Metabolism, Moffitt Cancer Center and Research Institute, Tampa, Florida USA; 7 Anatomic Pathology, Moffitt Cancer Center and Research Institute, Tampa, Florida USA; 8 Molecular Oncology, Moffitt Cancer Center and Research Institute, Tampa, Florida USA; 9 Department of Surgery, Division of General Surgery, University of Florida Health Sciences Center, Gainesville, Florida USA; 10 Department of Surgery, Sylvester Comprehensive Cancer Center at the University of Miami Miller School of Medicine, Miami, Florida USA

## Abstract

Pancreatic ductal adenocarcinoma (PDAC) is an aggressive disease that lacks effective biomarkers for early detection. We hypothesized that circulating long non-coding RNAs (lncRNAs) may act as diagnostic markers of incidentally-detected cystic PDAC precursors known as intraductal papillary mucinous neoplasms (IPMNs) and predictors of their pathology/histological classification. Using NanoString nCounter® technology, we measured the abundance of 28 candidate lncRNAs in pre-operative plasma from a cohort of pathologically-confirmed IPMN cases of various grades of severity and non-diseased controls. Results showed that two lncRNAs (*GAS5* and *SRA*) aided in differentiating IPMNs from controls. An 8-lncRNA signature (including *ADARB2*-*AS1*, *ANRIL*, *GLIS3*-*AS1*, *LINC00472*, *MEG3*, *PANDA*, *PVT1*, and *UCA1*) had greater accuracy than standard clinical and radiologic features in distinguishing ‘aggressive/malignant’ IPMNs that warrant surgical removal from ‘indolent/benign’ IPMNs that can be observed. When the 8-lncRNA signature was combined with plasma miRNA data and quantitative ‘radiomic’ imaging features, the accuracy of predicting IPMN pathological classification improved. Our findings provide novel information on the ability to detect lncRNAs in plasma from patients with IPMNs and suggest that an lncRNA-based blood test may have utility as a diagnostic adjunct for identifying IPMNs and their pathology, especially when incorporated with biomarkers such as miRNAs, quantitative imaging features, and clinical data.

## Introduction

Pancreatic ductal adenocarcinoma (PDAC) is the third leading cause of cancer deaths in the United States, with a five-year survival rate of only 9%^1^. Most cases are diagnosed at a late, incurable stage due to the lack of accurate methods for early detection^1^. Serum carbohydrate antigen 19-9 (CA19-9) is used to suggest a diagnosis of PDAC and monitor disease recurrence or response to therapy. However, it’s utility as a sensitive and specific marker of *early* PDAC is poor^2^. The detection and treatment of noninvasive precursor lesions offers hope in reducing PDAC-related morbidity and mortality. Intraductal papillary mucinous neoplasms (IPMNs) of the pancreas are a morphologically distinct set of tumors located in the duct epithelium and are characterized by papillary epithelial proliferation and mucin production, leading to cystic dilation of involved ducts^3^. These cystic PDAC precursor lesions (‘precancers’) comprise almost half of the ~150,000 asymptomatic pancreatic cysts detected incidentally in the general population each year by computed tomography (CT) scans and magnetic resonance imaging (MRI)^4, 5^. Despite their radiologic detection, the only way to accurately examine their severity which ranges from noninvasive/pre-malignant (low-grade (LG), moderate-grade (MG), or high-grade (HG) dysplasia) to invasive carcinoma is through surgical resection, which carries significant risks of morbidity and mortality^6–8^. Studies reveal that the 5-year survival rate for patients with noninvasive IPMNs is greater than 70%, which is significantly higher than the 22–45% rate for individuals with resected invasive IPMNs and the 10–25% survival rate reported for individuals with resected conventional PDACs^9^. Thus, proper diagnosis of disease and its severity (noninvasive versus invasive IPMN versus conventional PDAC not associated with an IPMN) is important for medical management.

Consensus guidelines for IPMN management^10^ depend on standard radiographic and clinical features and recommend that patients with ‘high risk stigmata (HRS)’ (main pancreatic duct (MD) involvement/dilatation >10 mm, obstructive jaundice with a cystic lesion in the pancreatic head, or an enhanced solid component/nodule within the cyst) undergo resection, as most harbor high-grade or invasive disease^10^. More challenging to manage are IPMNs having ‘worrisome features (WF)’ (MD dilation 5–9 mm, cyst size >3 cm, thickened enhanced cyst walls, non-enhanced mural nodules, or acute pancreatitis). It is suggested that these lesions undergo endoscopic evaluation with endoscopic ultrasound with fine needle aspiration (EUS-FNA) despite suboptimal sensitivity and technical complications^5, 11^. The guidelines provide an important framework for management, but there is disagreement between the preoperative diagnosis and pathologic examination in a large percentage (30–70%) of cases^10, 12–16^. Novel, noninvasive markers of IPMN pathology are needed, especially for individuals who do not present with HRS.

Non-coding RNAs (ncRNAs) are outstanding candidate biomarkers of early neoplasia due to their stability in tissue^17, 18^ and biofluids^19, 20^ and their ability to regulate hundreds of genes and biological pathways. ncRNAs are typically classified according to their size; small ncRNAs are less than 200 nucleotides in length whereas long ncRNAs contain at least 200 nucleotides and resemble protein-coding transcripts but without functional open reading frames^21^. We^22, 23^ and others^24–26^ have conducted genome-wide analysis of miRNAs, the best characterized class of small ncRNAs, using tissue and plasma or serum from IPMN patients and healthy controls. Collectively, these data support miRNAs as a promising diagnostic adjunct for stratifying IPMN patients for surveillance or resection. Furthermore, in a small retrospective cohort of IPMN cases, we found diagnostic performance to improve when combining plasma miRNA data with quantitative ‘radiomic’ features invisible to the human eye extracted from routine CT scans^27^. LncRNAs are not as well studied as miRNAs even though they are the predominant transcribed RNAs^28^ and have been shown to regulate gene expression and promote carcinogenesis through mechanisms such as transcriptional regulation, initiation of chromatin remodeling, modulating alternative splicing, altering protein activity or localization, and genomic imprinting^21, 29, 30^. Given emerging data on the role of lncRNAs in PDAC initiation, progression, and outcomes^31–39^ and evidence to support lncRNA detection in circulation^38, 40–44^, we conducted the first study to measure the abundance of circulating lncRNAs from patients with IPMNs.

Here we quantified the abundance of 28 candidate lncRNAs in archived plasma obtained pre-operatively from individuals with noninvasive and invasive IPMNs and age- and gender-matched disease-free controls using the nCounter® technology (NanoString, Inc, Seattle, Washington) digital quantification method^45^. We then aimed to (1) discover circulating lncRNAs that may distinguish between patients with IPMNs and non-diseased controls, (2) identify circulating lncRNAs that may differentiate malignant/aggressive IPMNs (classified as those pathologically confirmed to have high-grade dysplasia or invasive disease) from benign/more indolent IPMNs (classified as those confirmed to have low- or moderate-grade dysplasia), and (3) determine the performance of circulating lncRNAs in predicting IPMN pathology individually and in combination with existing miRNA and quantitative imaging data described in our previous publications^22, 27^. Our findings provide new information on circulating lncRNAs and their potential to help individualize risk assessment and management for individuals with IPMNs.

## Results

### Study Population

Pre-operative plasma samples were evaluated for 57 IPMN cases and 24 non-diseased controls frequency-matched by age-group and gender. Eight samples were excluded prior to normalization and statistical analysis due to: failure to amplify (1 case) and high binding density due to possible over-seeding of the nCounter cartridge or erythrocyte contamination (5 cases, 2 controls), leaving samples from 73 participants (51 cases, 22 controls) for analysis. Study population characteristics are shown in Table 1. Cases and controls were well-matched on age (mean age: 68.5 vs 68.2 years). Most subjects were white, non-Hispanic. IPMN cases were more likely (47%) to have smoked cigarettes than controls (36%). The distribution of low-, moderate-, high-grade, and invasive IPMN cases was 12%, 29%, 24%, and 35%, respectively. Selected characteristics of the IPMN cohort are summarized in Table 2. Pre-operatively, compared to benign IPMNs (those with pathologically-confirmed low- or moderate grade dysplasia), malignant IPMNs (those with high-grade or invasive disease) were significantly (p < 0.05) more likely to be associated with: jaundice as a presenting symptom, tumors in the pancreatic head, MD involvement, and a solid component or mural nodule.

**Table 1:** Characteristics of the Study Population (N = 73).

Variable	IPMN cases (n = 51)	Healthy controls (n = 22)
Age at diagnosis/interview, mean (SD)(yrs)	68.5 (10.0)	68.2 (9.4)
Gender, male: female, n (%)	27 (53): 24 (47)	11 (50): 11 (50)
Race, n (%)		
White, Non-Hispanic	47 (92)	22 (100)
Other	4 (8)	0 (0)
Ever Smoker, n (%)		
Yes	24 (47)	8 (36)
No	21 (41)	8 (36)
Unknown	6 (12)	6 (27)
IPMN Grade, n (%)		
Low	6 (12)	—
Moderate	15 (29)	—
High	12 (24)	—
Invasive	18 (35)	—

Data represent counts (percentages) unless otherwise indicated. Counts may not add up to the total due to missing values, and percentages may not equal 100 due to rounding.

**Table 2:** Characteristics of IPMN cases in the cohort (N = 51).

Variable	Benign^1^ IPMNs (n = 21)	Malignant^2^ IPMNs (n = 30)	*p*value
Age at diagnosis, mean (SD)(yrs)	68.4 (9.8)	68.6 (10.3)	0.939
Male: Female, n (%)	8(38):13(62)	19(63):11(37)	0.075
Body mass index (BMI), mean (SD)	26.5 (4.6)	27.9 (4.6)	0.355
Positive personal history of diabetes	4 (19)	4 (13)	0.621
Positive personal history of chronic pancreatitis	5 (24)	9 (30)	0.126
Had abdominal pain as presenting symptom	7 (37)	11 (37)	0.165
Had weight loss as presenting symptom	3 (14)	8 (27)	0.110
Had jaundice as presenting symptom	1 (5)	8 (27)	**0**.**012**
Pre-operative serum CA 19-9 levels, mean (SD)(ng/mL)	91 (314)	692 (1493)	0.125
Pre-operative serum albumin levels, mean (SD)(ng/mL)	4.4 (0.98)	3.9 (0.66)	0.073
Predominant tumor location			
Pancreatic Head	6 (29)	14 (47)	
Pancreatic Body or Tail	14 (67)	12 (40)	**0**.**013**
Diffuse	1 (5)	4 (13)	
Type of ductal communication			
Main duct or mixed	4 (22)	10 (30)	**0**.**003**
Branch duct	14 (78)	4 (13)	
Size of largest cyst on imaging, mean (range) (cm)	2.8 (1.6)	3.5 (1.4)	0.145
Solid component or mural nodule			
Yes	3 (14)	8 (27)	
No	15 (71)	6 (20)	**0**.**021**

Data represent counts (percentages) unless otherwise indicated. Counts may not add up to the total due to missing values, and percentages may not equal 100 due to rounding.

^1^Benign IPMNs are represented by 6 low-grade and 15 moderate-grade IPMNs.

^2^Malignant IPMNs are represented by 12 high-grade and 18 invasive IPMNs.

### Analysis of plasma lncRNAs in IPMN cases versus non-diseased controls

Each of the 28 lncRNAs had signals that were detectable ﻿above background and could be included in analyses. No statistically significant differences were observed between IPMN and control samples in the frequency or the amount of erythrocyte contamination. After normalization, two lncRNAs (*GAS5* and *SRA*) differentiated the 51 IPMN samples from the 22 control samples using a threshold of p < 0.05. Compared to controls, IPMN cases had 0.9-fold lower *GAS5* expression and 1.2 fold higher *SRA* expression (Supplementary Figure S1). The 2-lncRNA signature, represented by PC1, explained 60% of the variability in the data, suggesting it represents the signature well. The area under the curve (AUC) value was 72.9 (95% CI: 60.9–84.9). The sensitivity, specificity, positive predictive value (PPV), and negative predictive value (NPV) for IPMN detection were 82%, 59%, 82% and 59%, respectively. Expression results for cases and controls are in Supplementary Table S1.

### Analysis of plasma lncRNAs in malignant versus benign IPMNs

**Figure 1:**
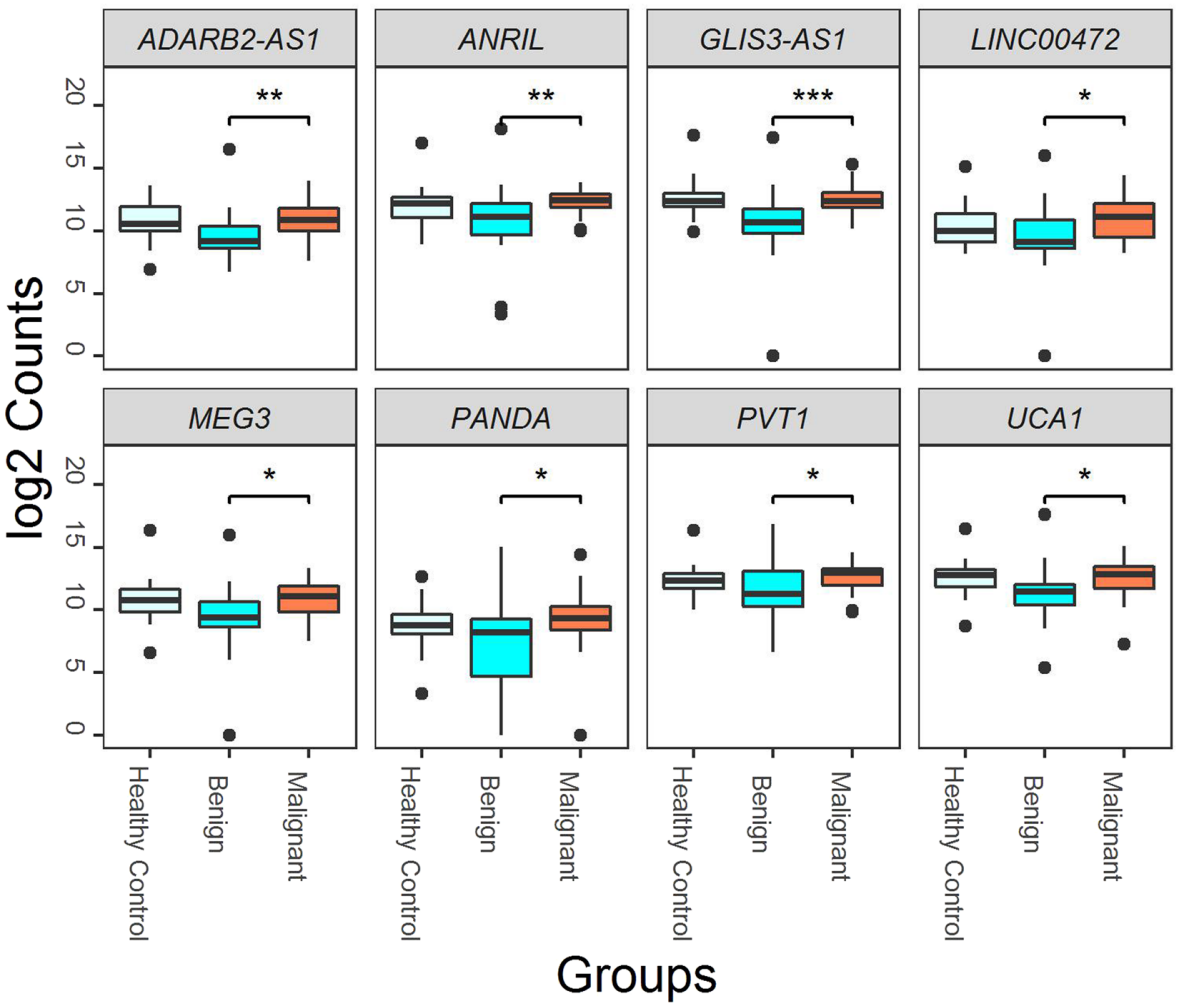
Eight lncRNAs in circulation discriminated malignant (n = 30) from benign (n = 21) IPMN cases (*p < 0.05, **p < 0.01 and ***p < 0.001)). Box plots displaying the distribution of the abundance of each individual lncRNA within the malignant and benign groups and among healthy controls.

**Figure 2:**
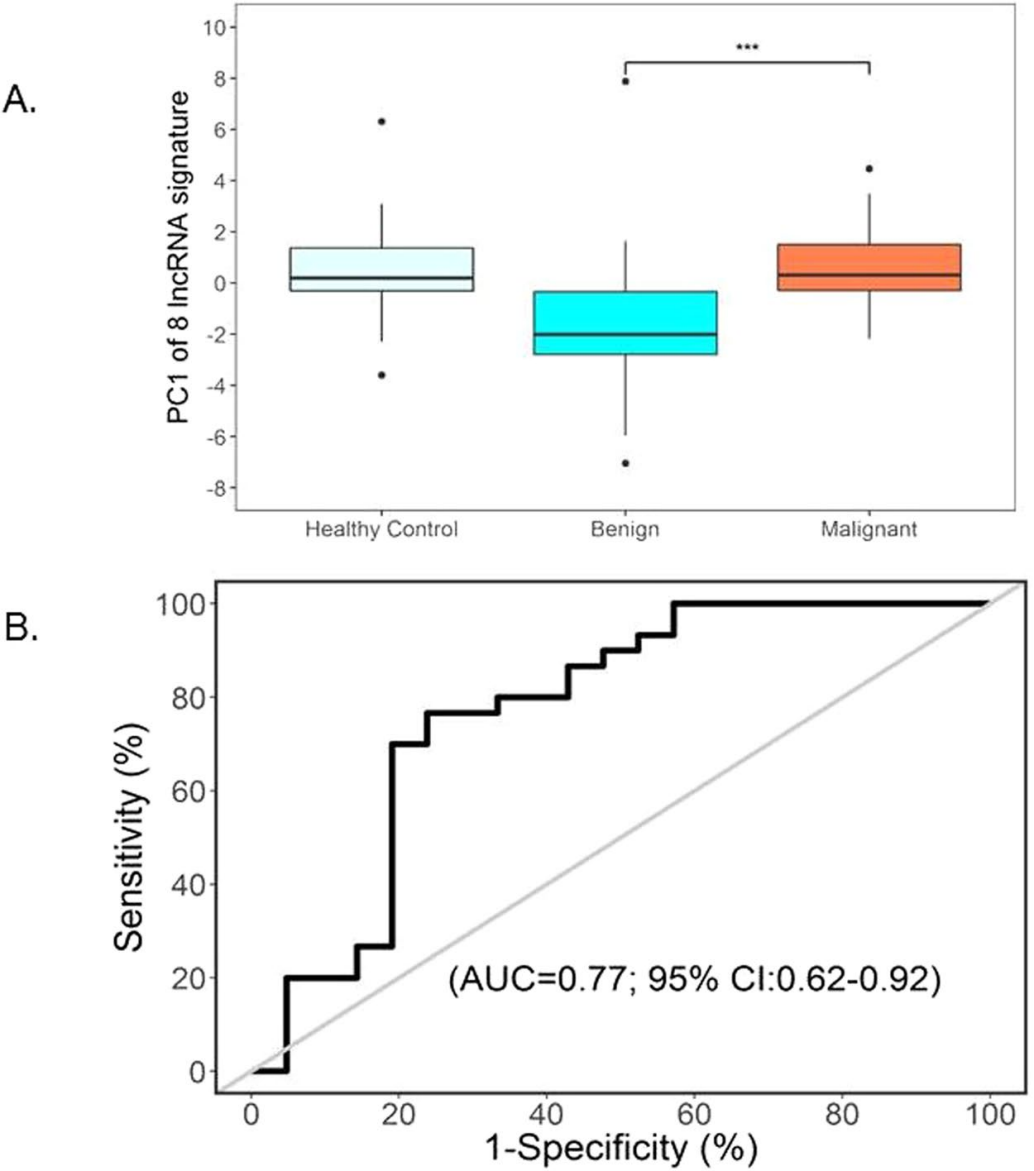
The 8-lncRNA signature associates with IPMN pathology. (**A**) Box plots of the distribution of the 8-lncRNA signature score (designated by the first principal component (PC1)) within the malignant and benign groups and healthy controls. (**B**) Receiver operating characteristic (ROC) curve analysis showed that the 8-lncRNA signature PC1 yielded an area under the curve (AUC) value of 0.77 (95% CI: 0.62–0.92) in differentiating between malignant and benign IPMN groups.

**Figure 3:**
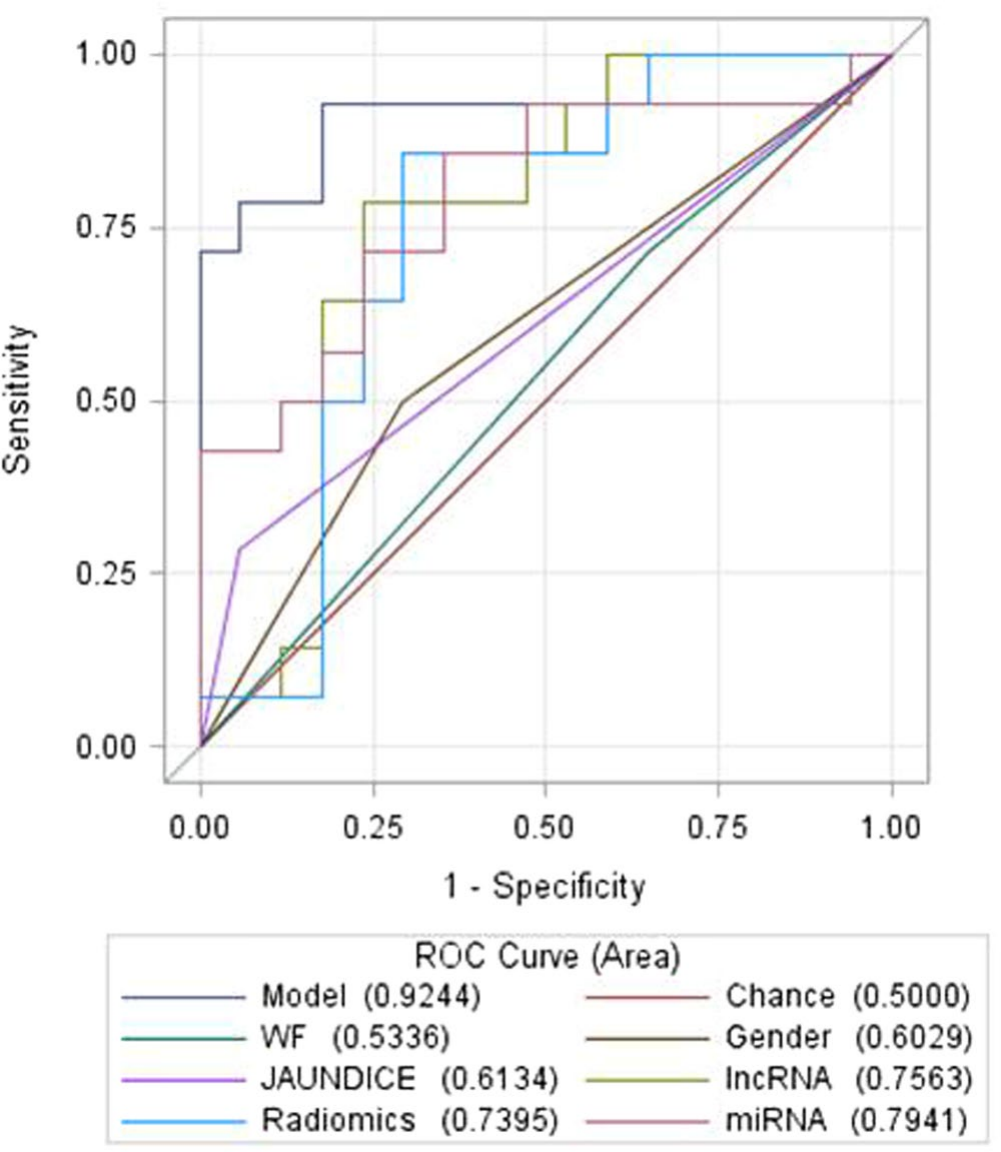
ROC analysis suggests that genomic data (the 8-lncRNA signature and a 5-miRNA signature) and quantitative radiomic features are more accurate in predicting malignant IPMN pathology than standard worrisome radiologic features and certain demographic and clinical variables. A final model combining the 8-lncRNA signature, the 5-miRNA signature, radiomic features, standard worrisome features (WF), gender, and presence of jaundice has potential to have high accuracy in predicting malignant pathology, with an AUC value approximating 0.92.

We evaluated the ability of the 2-lncRNA signature (identified in the analysis of IPMN cases versus non-diseased controls) to discriminate between the 30 malignant and 21 benign IPMNs and observed it did not perform well (AUC = 55.1 (95% CI: 38.1–72.1)). However, a focused analysis of the 51 IPMN cases showed that a signature of eight lncRNAs (*ADARB2*-*AS1*, *ANRIL*, *GLIS3*-*AS1*, *LINC00472*, *MEG3*, *PANDA*, *PVT1*, and *UCA1*) discriminated between the malignant and benign IPMNs (p < 0.05) (Table 3; Fig. 1). The 8-lncRNA signature was also characterized by a PC1 score and explained 67% of the variability. The overall expression of the 8-lncRNA signature was higher in malignant compared to benign IPMNs (p = 0.001, Fig. 2), was significantly associated with malignant status (OR (95% CI): = 1.56 (1.11–2.21), p = 0.011), and had an AUC value of 0.77 (95% CI: 0.62–0.92) in discriminating between groups (Fig. 2). Estimates of sensitivity, specificity, PPV, and NPV of the 8-lncRNA signature in discriminating malignant from benign IPMNs were 77%, 76%, 82%, and 70%, respectively. The 8-lncRNA signature also had a high specificity (91%) and PPV (90%) when comparing all IPMN cases to healthy controls. However, when comparing only malignant IPMN cases to healthy controls, neither individual lncRNAs or the 8-lncRNA signature clearly differentiated between these two groups (Fig 2 and 3), suggesting further interrogation will be necessary in larger datasets.

**Table 3:** LncRNA expression in malignant (n = 30) versus benign (n = 21) IPMN cases.

LncRNA	Overall mean	Benign mean	Malignant mean	p value	False discovery rate	Fold Change
*GLIS3-AS1*	11.7	10.6	12.4	0.005	0.120	1.2
*ANRIL*	11.7	10.7	12.3	0.009	0.120	1.2
*PANDA*	8.4	7.0	9.3	0.016	0.141	1.3
*MEG3*	10.3	9.4	10.9	0.022	0.141	1.2
*PVT1*	12.2	11.6	12.6	0.037	0.141	1.1
*ADARB2-AS1*	10.4	9.8	10.8	0.039	0.141	1.1
*LINC00472*	10.4	9.6	10.9	0.039	0.141	1.1
*UCA1*	12.1	11.4	12.6	0.041	0.141	1.1
*PPP3CB*	13.5	13.1	13.8	0.058	0.176	1.1
*LINC00469*	9.6	8.7	10.3	0.099	0.277	1.2
*LINC00491*	9.9	9.2	10.5	0.133	0.337	1.1
*TERC*	9.2	8.7	9.5	0.153	0.355	1.1
*AS1DHRS4*	13.4	13.0	13.7	0.183	0.365	1.0
*MALAT1*	10.7	10.4	11.0	0.198	0.365	1.1
*H19*	15.0	14.6	15.2	0.206	0.365	1.0
*PTENP1*	6.5	5.8	6.9	0.209	0.365	1.2
*GAS5*	15.2	14.9	15.4	0.240	0.379	1.0
*HOXD-AS1*	7.1	6.6	7.4	0.245	0.379	1.1
*BCYRN1*	0.2	0.4	0.1	0.336	0.506	0.1
*HULC*	2.1	1.5	2.5	0.395	0.531	1.6
*HOTAIR*	6.5	6.3	6.7	0.400	0.531	1.1
*XIST*	0.9	0.7	1.1	0.537	0.683	1.6
*LINC00244*	0.9	0.7	1.0	0.594	0.690	1.4
*SRA*	5.5	5.4	5.6	0.624	0.690	1.0
*DDX6P*	2.9	2.6	3.1	0.632	0.690	1.2
*lncRNA-p21*	5.9	5.7	6.0	0.642	0.690	1.0
*HOTTIP*	5.8	5.6	6.0	0.668	0.693	1.1
*aHIF*	9.6	9.6	9.5	0.849	0.849	1.0

### Relationship between standard clinical and radiologic data, IPMN pathology, and lncRNA expression

Clinical factors associated (p < 0.05) with an increased risk of malignancy included cyst size greater >3 cm (OR (95% CI) = 2.60 (0.76–8.86, p = 0.127), main duct involvement (OR (95% CI) = 11.00 (2.26–53.63, p = 0.003), and serum albumin levels (OR (95% CI) = 0.35 (0.11–1.15, p = 0.083). Cyst size positively correlated with the 8-lncRNA signature (Spearman correlation = 0.243, p = 0.125), while serum albumin levels negatively correlated with the 8-lncRNA signature (Spearman correlation = −0.158, p = 0.356). Mean serum CA 19-9 levels were higher in the malignant compared to benign group, but the difference was not statistically significant (p = 0.130). Of the identified lncRNAs, only *PANDA* was associated with serum CA 19-9 levels (p = 0.018), but the spearman correlation coefficient was not strong (r = 0.41). The 8-lncRNA signature was independently associated with malignant status after adjustment for MD involvement (p = 0.033) and presence of jaundice (p = 0.0095).

### Combining lncRNA data with miRNA and radiomic data

Our group recently evaluated the possibility of using a multimodal approach to predict IPMN pathology that combines a 5-miRNA signature and a 14-feature radiomic CT signature generated using previously described procedures^27^ (see methods). We showed that a model which integrates miRNA and radiomic data had an area under the curve (AUC) value of 0.92 in discriminating 18 malignant from 20 benign cases; this was far superior to the AUC value for standard radiologic worrisome features (AUC = 0.54). In the current investigation, we integrated the 8-lncRNA signature with the 5-miRNA signature and the 14-feature radiomic CT signature and re-assessed diagnostic performance.

Of the 51 IPMN cases represented in the current investigation of lncRNAs, 31 (14 malignant, 17 benign) have available paired pre-operative plasma miRNA data and CT data. As summarized in Table 4, diagnostic performance increased from an AUC value of 0.76 when considering the 8-lncRNA signature alone to 0.90 when combining the 8-lncRNA signature, the 5-miRNA signature, and the 14-feature radiomic signature due to an increase in specificity. Finally, by incorporating standard radiologic features (worrisome features in the absence of high risk stigmata), gender, and presence of jaundice, the AUC increased to 0.92 (95% CI: 0.82–1.00) and revealed estimates of sensitivity, specificity, PPV and NPV of 93%, 82%, 81%, and 93%, respectively (Fig. 3 and Table 4). Importantly, the integrated discrimination improvement (IDI) index significantly increased with the addition of the 8-lncRNA signature, the 5-miRNA signature, and the radiomics signature, with respective IDI indices of 0.12, 0.14, and 0.13. Collectively, when the two molecular signatures and the radiomic signature were added to the model containing WF, gender, and jaundice, the IDI increased to 0.41 (95% CI: 0.23–0.59), p < 0.001. As anticipated, models that considered presence of high-risk stigmata individually or in conjunction with other data types performed well.

**Table 4:** Diagnostic performance of preliminary models to predict malignant IPMN pathology^1^.

Model/Variables included	AUC (95% CI)	p value	FDR	Accuracy	SE	SP	PPV	NPV
Gender	0.60 (0.43 ~ 0.78)	0.242	0.252	0.61	0.50	0.71	0.58	0.63
Jaundice	0.61 (0.48 ~ 0.75)	0.087	0.095	0.65	0.29	0.94	0.80	0.62
High risk stigmata (HRS)	0.84 (0.71 ~ 0.97)	**0**.**0002**	**0**.**004**	0.84	0.86	0.82	0.80	0.88
Worrisome features (WF)	0.53 (0.36 ~ 0.70)	0.690	0.690	0.52	0.71	0.35	0.48	0.60
lncRNA signature	0.76 (0.58 ~ 0.94)	**0**.**012**	**0**.**022**	0.77	0.79	0.76	0.73	0.81
miRNA signature	0.79 (0.63 ~ 0.96)	**0**.**035**	**0**.**043**	0.74	0.86	0.65	0.67	0.85
Radiomics signature	0.74 (0.55 ~ 0.93)	**0**.**014**	**0**.**023**	0.77	0.86	0.71	0.71	0.86
miRNAs + Radiomics	0.89 (0.76 ~ 1.00)	**0**.**002**	**0**.**009**	0.87	0.79	0.94	0.92	0.84
lncRNAs + Radiomics	0.78 (0.61 ~ 0.95)	**0**.**010**	**0**.**021**	0.81	1.00	0.65	0.70	1.00
lncRNAs + miRNAs	0.83 (0.67 ~ 0.99)	**0**.**013**	**0**.**023**	0.81	0.79	0.82	0.79	0.82
lncRNAs + Radiomics + miRNAs	0.90 (0.78 ~ 1.00)	**0**.**004**	**0**.**010**	0.87	0.71	1.00	1.00	0.81
HRS + WF + lncRNAs	0.91 (0.81 ~ 1.00)	**0**.**001**	**0**.**007**	0.87	0.86	0.88	0.86	0.88
HRS + WF + Radiomics	0.85 (0.70 ~ 1.00)	**0**.**002**	**0**.**009**	0.84	0.86	0.82	0.80	0.88
HRS + WF + miRNAs	0.94 (0.87 ~ 1.00)	**0**.**001**	**0**.**007**	0.87	0.93	0.82	0.81	0.93
HRS + WF + lncRNAs + Radiomics	0.91 (0.81 ~ 1.00)	**0**.**003**	**0**.**009**	0.87	0.86	0.88	0.86	0.88
HRS + WF + lncRNAs + miRNAs	0.95 (0.89 ~ 1.00)	**0**.**001**	**0**.**007**	0.87	0.93	0.82	0.81	0.93
HRS + WF + lncRNAs + Radiomics + miRNAs	0.95 (0.89 ~ 1.00)	**0**.**003**	**0**.**009**	0.87	0.93	0.82	0.81	0.93
HRS + WF + gender + Jaundice + lncRNAs + Radiomics + miRNAs	0.97 (0.91 ~ 1.00)	**0**.**010**	**0**.**021**	0.90	0.93	0.88	0.87	0.94
WF + lncRNAs	0.76 (0.59 ~ 0.94)	**0**.**041**	**0**.**049**	0.77	0.71	0.82	0.77	0.78
WF + Radiomics	0.78 (0.60 ~ 0.95)	**0**.**021**	**0**.**031**	0.81	1.00	0.65	0.70	1.00
WF + miRNAs	0.79 (0.62 ~ 0.96)	0.067	0.076	0.81	0.79	0.82	0.79	0.82
WF + lncRNAs + Radiomics	0.82 (0.66 ~ 0.97)	**0**.**015**	**0**.**024**	0.77	0.86	0.71	0.71	0.86
WF + lncRNAs + miRNAs	0.84 (0.69 ~ 1.00)	**0**.**029**	**0**.**039**	0.84	0.86	0.82	0.80	0.88
WF + lncRNAs + Radiomics + miRNAs	0.92 (0.83 ~ 1.00)	**0**.**007**	**0**.**018**	0.87	0.93	0.82	0.81	0.93
WF + gender + Jaundice + lncRNAs + Radiomics + miRNAs	0.92 (0.82 ~ 1.00)	**0**.**027**	**0**.**037**	0.87	0.93	0.82	0.81	0.93

^1^31 IPMN cases (17 benign; 14 malignant) had data types (clinical data, miRNA, radiomic, lncRNA) included in these analyses. AUC = area underneath the curve; SE = sensitivity; SP = specificity; PPV = positive predictive value; NPV = negative predictive value; High risk stigmata = main pancreatic duct involvement/dilatation ≥10 mm, obstructive jaundice with a cystic lesion in the pancreatic head, or an enhanced solid component/nodule within the cyst; Worrisome features = main pancreatic duct dilation 5–9 mm, cyst size >3 cm, thickened enhanced cyst walls, non-enhanced mural nodules, or acute pancreatitis.

### Cross-validation analysis

Evaluation of uncertainty by 10-fold cross-validation showed fairly robust estimates of diagnostic performance with AUC above 0.70 for most models that incorporated lncRNAs (Supplementary Table S2). A model that combined lncRNAs, miRNAs, radiomics, and worrisome features had an AUC = 0.77 (95% CI: 0.68–0.84) in cross-validation and was more accurate than standard demographic (gender), clinical (presence of jaundice), and radiologic variables (worrisome features) in predicting malignant pathology.

## Discussion

This is the first investigation to report on lncRNA quantification using plasma from individuals newly-diagnosed with commonly detected PDAC precursors known as IPMNs. nCounter technology® was used to measure lncRNAs as an alternative to microarray and PCR-based methods to more accurately detect and quantify low lncRNA levels present in blood^46^. Similar to our previous study of circulating miRNAs^22^, an extensive quality control and data analysis pipeline was implemented to control for pre-analytical and technical factors that may affect circulating lncRNA levels and result in biases that do not reflect underlying biology. We show that lncRNAs can be detected in plasma, and provide data to support the possibility that this class of noninvasive biomarkers may serve as an adjunct to help predict IPMN severity/pathology.

To evaluate whether lncRNAs may serve as diagnostic markers of PDAC, recent studies^37, 39, 47–50^ have evaluated lncRNA expression in PDAC tumor tissue and adjacent normal tissue, and a few^51, 52^ have evaluated lncRNA expression in the circulation of PDAC patients, control patients, and healthy individuals using blood^51, 53^ or saliva^52^. Of the three biofluid-based studies^51, 52^, only one^51^ focused on detection of early-stage PDAC and none studied PDAC precursors. Hence, to our knowledge, we are unaware of previous published reports of lncRNA signatures derived from tissues, blood, or other biofluids from patients with IPMNs. Using plasma, we show that two lncRNAs (*GAS5* and *SRA*) can partially discriminate IPMN cases from non-diseased controls (AUC) = 0.73 (95% CI:0.61–0.85, p = 0.004). *GAS5* (growth arrest-specific 5) was found to have *lower* expression in IPMN cases compared to controls (p = 0.028), consistent with data^33^ showing that *GAS5* expression is significantly *decreased* in pancreatic cancer tissues compared with normal pancreatic tissues. The authors^33^ demonstrate that *GAS5* inhibition induces a significant decrease in G0/G1 phase and an increase in S phase, whereas overexpression in PDAC cells inhibits cell proliferation by negatively regulating *CDK6* (cyclin-dependent kinase 6) expression *in vitro* and *in vivo*. Furthermore, a recent study showed that decreased *GAS5* levels in serum were associated with type 2 diabetes in a cohort of US military veteran^54^, suggesting this lncRNA may help to identify individuals ‘at-risk’ for diabetes. *GAS5* expression was not associated with presence of diabetes among IPMN cases in our small dataset (p = 0.36), but given that diabetes is an established risk factor for PDAC and ‘new-onset’ diabetes may serve as a potential marker of early PDAC^55^, further research may be indicated to explore a possible role for *GAS5* in the molecular pathogenesis of diabetes-mediated PDAC. *SRA* (steroid receptor RNA activator) is responsible for coordinating functions of transcription factors and enhancing steroid receptor-dependent gene expression^56^. Specifically, as a nuclear receptor coactivator, *SRA* can coactivate androgen receptor (AR), estrogen receptor alpha (ERalpha), ERbeta, progesterone receptor (PR), glucocorticoid receptor (GR), thyroid hormone receptor and retinoic acid receptor (RAR). Emerging studies have revealed that SRA plays a key role in biological processes (such as myogenesis and steroidogenesis) and pathological changes (such as obesity and tumorigenesis)^56, 57^. Thus, it is biologically plausible *SRA* could be expressed at higher levels in IPMNs compared to normal controls. Moreover, in our dataset, patients with malignant IPMNs had higher levels of *SRA* than those with benign IPMNs. Further investigation is needed in larger cohorts before conclusions can be reached.

Analysis also revealed an 8-lncRNA signature (comprising *ADARB2*-*AS1*, *ANRIL*, *GLIS3*-*AS1*, *LINC00472*, *MEG3*, *PANDA*, *PVT1*, and *UCA1*) that partially discriminates between malignant and benign IPMNs AUC = 0.77 (95% CI: 0.62–0.92, p = 0.006). These lncRNAs were upregulated in malignant compared to benign cases. Consistent with our findings, several of the identified lncRNAs (*UCA1*, *PVT1*) appear to have oncogenic functions in pancreatic carcinogenesis^47, 48, 52, 53^, inferring biological plausibility of the 8-lncRNA signature. Urothelial cancer-associated 1 (*UCA1*) is known for its role in bladder cancer progression and embryologic development, and in two separate investigations^47, 48^ has recently been shown to be upregulated in PDAC tissues (versus matched adjacent normal pancreas tissue), to be associated with several prognostic factors (such as stage, tumor size, and grade), and shorter survival. Furthermore, functional experiments have shown that *UCA1* promotes invasion and proliferation of PDAC cells and that down-regulation of *UCA1* inhibits cell proliferation and induces apoptosis and cell cycle arrest^47, 48^. *PVT1* (plasmacytoma variant translocation 1) has been shown to be upregulated in PDAC tissues, to be correlated with clinical stage and poor survival, to be overexpressed in the saliva of PDAC cases versus healthy controls, and to contribute to susceptibility to PDAC as part of a genome-wide association study^53, 54, 58^. Furthermore, *PVT1* has been identified as a regulator of gemcitabine sensitivity; *PVT1* inactivation led to enhanced gemcitabine sensitivity in human PDAC cells^59^. Thus, in addition to serving as prognostic markers and therapeutic targets, it is biologically plausible that *UCA1* and *PVT1* may also contribute to early pancreatic carcinogenesis (and malignant potential) as observed in this study. *ANRIL* (antisense non-coding RNA in the INK4 locus) was also shown to have higher expression in malignant versus benign IPMNs. Although we are unaware of reports of ANRIL dysregulation and PDAC, increased expression of this well-known lncRNA has been shown to contribute to the risk of diabetes^60^, a risk factor for PDAC^55^ and IPMNs^61^.

Several of the lncRNAs highlighted in our investigation appear to have a tumor suppressor role in pancreatic neoplasms. For example, maternally expressed gene 3 (*MEG3*) was recently shown to inhibit PDAC proliferation via activation of p53 and to play a key role in the anti-tumor effects of fenofibrate, a PPAR-α agonist^62^. Additionally, epigenetic activation of *MEG3* (and inactivation of its target c-MET) with DNA methylating drugs has been shown to have a therapeutic effect on pancreatic neuroendocrine tumors^63^. Analyses in breast^64^ and ovarian tumors^65^ support a tumor suppressor role for intergenic lncRNA, *LINC00472*, but functional investigations of this gene are lacking. A recent phenome-genome association study of pancreatic cancer^66^ revealed several open reading frames associated with pancreatic neoplasms, and included candidates *LINC00472*, *GLI3*-*AS1*, *and ADARB2*-*AS1*. Finally, although reports of the candidate lncRNA *PANDA* (P21associated noncoding RNA DNA damage-activated) and PDAC are lacking, data suggest it plays a role in stabilizing p53 in response to DNA damage^67^ and in regulating senescence^68^.

To increase diagnostic performance, we combined the 8-lncRNA signature with a 5-miRNA plasma signature, ‘radiomic’ imaging features, and clinical characteristics available through previous studies^22, 27^. We showed that integration of multiple data types improves prediction of IPMN pathology beyond that provided by standard clinical and radiologic characteristics, especially worrisome features considered in consensus guidelines^10^. Although model overfitting may have contributed to our findings and warrants further interrogation in larger datasets, results of the integrated discrimination improvement (IDI) test and the cross-validation analysis suggest that these novel data types do have potential to add value in IPMN risk assessment.

Despite the strengths and novelty of this study, there are limitations that merit consideration. Given that lncRNAs are typically expressed at low concentrations in circulation, we used the robust nCounter platform (as opposed to quantitative real time RT-PCR as others have done) and performed a pre-amplification procedure at the suggestion of the manufacturer and available literature. Although pre-amplification has potential to introduce amplification bias or assay cartridge saturation, head-to-head comparisons of plasma samples with and without pre-amplification have shown a higher level of linearity in samples with pre-amplification^40^. Furthermore, circulating lncRNAs have been shown to be stable under different experimental conditions^40^. Although mechanisms accounting for lncRNA stability are incompletely understood, data suggest they may be protected by exosome encapsulation and/or complex formation with proteins and miRNAs^38, 69^. Thus, integrity of our findings may not have been significantly influenced by pre-amplification or sample instability. Our study is also limited in that we evaluated only 28 candidate lncRNAs. It is possible that lncRNAs not evaluated here (such as Linc-pint, a lncRNA observed in plasma to be a possible biomarker of early PDAC by Li *et al*.^51^) could be important to IPMN pathogenesis.

Other limitations of the current study are that we were unable to examine post-operative levels of plasma lncRNAs to see if they normalize following the removal of the lesion and we were unable to evaluate whether IPMN tissue is the origin of the circulating lncRNAs. Plasma is now being collected and processed pre- *and* post-surgery and during surveillance ﻿as par﻿t of ou﻿r multi-institutional cohort study known as the Florida Pancreas Collaborative^70^ so that we can assess changes in biomarker levels over time. Due to the small or focal nature of IPMNs and the lack of ample tissue for molecular analyses for most cases, tissue microarrays (rather than whole sections) are being created to perform *in situ* hybridization for the most promising lncRNAs. Finally, the relatively small sample size of our study population limits the ability to draw meaningful conclusions. External validation in a large, multi-center prospective investigation of serial plasma lncRNA measurements is indicated for individuals newly-diagnosed with various types of pancreatic cysts and early-stage PDAC, and those at high genetic risk for developing PDAC, and healthy individuals without cancer.

In summary, lncRNAs can be detected in plasma and have potential to be incorporated clinically as part of a blood-based diagnostic adjunct to aid in IPMN management, especially in conjunction with other types of biomarkers (such as miRNAs) and quantitative radiologic features. Large-scale studies with rigorous designs and incorporation of epidemiologic and clinical data are needed to further explore the potential for circulating lncRNAs to be utilized as novel biomarkers for IPMN diagnosis and monitoring and as targets for intervention using RNA interference (RNA-i)-mediated approaches.

## Materials and Methods

### Study population and biospecimens

A prospectively maintained clinical database was retrospectively reviewed to identify individuals who underwent a pancreatic resection for an IPMN between 2006 and 2011 at Moffitt Cancer Center and Research Institute (Moffitt) and had provided written consent for blood to be donated pre-operatively for research through protocols approved by the Institutional Review Board (IRB) of the University of South Florida, including Total Cancer Care^71^. IRB approval was granted for study participation and the research described herein (IRB#Pro4971), written informed consent was obtained from study participants, and all methods were performed in accordance with relevant guidelines and regulations. The diagnosis and degree of dysplasia was pathologically confirmed using World Health Organization (WHO) guidelines^3^. The final diagnosis represented the most severe grade of dysplasia observed in the neoplastic epithelium. None of the included cases received pre-operative chemotherapy or radiation. Also eligible for inclusion were age- and gender- matched healthy controls with no current or prior history of pancreatic disease or symptoms who presented to Moffitt’s Cancer Screening and Prevention Center during the same time period and donated blood through a related IRB-approved protocol using the same procedures.

Blood was collected from consented participants via phlebotomy in a 7-mL EDTA tube and processed for plasma within two hours using standard procedures^72^. The tube was inverted 3 times and spun at 3600 rpm for 8 minutes and then aliquoted into 0.5 mL bar-coded cryovials and banked at −80 °C. Demographic, clinical, and epidemiologic data was collected from an electronic questionnaire, the medical record, Moffitt’s cancer registry, and other sources.

### RNA isolation and multiplexed target enrichment (MTE)

One 0.5 mL cryovial of plasma was retrieved and thawed for each study participant. To assess hemolysis, samples were visually inspected and spectrophotometric analysis was performed at 414, 541, and 576 nm^73^. Samples were classified as hemolyzed if the A_414_, A_541_, or A_576_ value exceeded 0.2. Synthetic spike-in RNA representing NEFL (neurofilament 1), ENO2 (neuron-specific endonuclease) and GFAP (glial fibrillary acidic protein) were added to plasma to control for variance in the starting material and the efficiency of RNA extraction, according to vendor recommendations. Cell-free RNA was then isolated from 500 µl of plasma using the Plasma/Serum RNA Purification Midi Kit with a final elution volume of 50 µl (Norgen Biotek Corp, Ontario, CA). The extracted RNA was further purified and concentrated down to 30 µl using the Zymo RNA Clean and Concentrator-5 kit (Zymo Research Corp, Irvine, CA). The concentrated RNA was qualitatively assessed on the Agilent BioAnalyzer Total RNA Pico chip (Agilent Technologies, Santa Clara, CA). As the RNA was expected to be degraded, a BioAnalyzer peak from 25–200 nt indicated successful RNA recovery. Four µl of RNA was reverse transcribed into single-stranded cDNA using the Invitrogen SuperScript VILO cDNA Synthesis Kit and Master Mix (Thermo Fisher Scientific, Waltham, MA) following the nCounter Single Cell Gene Expression protocol (NanoString Technologies, Seattle, WA). The single-stranded cDNA was enriched using a highly multiplexed pool of target-specific PCR primer pairs targeting 28 lncRNAs. The MTE process was carried out using 22 cycles of PCR using the ABI TaqMan PreAmp MasterMix (Thermo Fisher Scientific, Waltham, MA) with the conditions described in the NanoString protocol.

### Quality control of the multiplexed target enrichment and hybridization

The resulting PCR products from the Multiplexed Target Enrichment were assessed using quantitative RT-PCR (qPCR) to evaluate the expression of several target genes and normalize the amount of MTE DNA input into the NanoString hybridization. The MTE PCR products were diluted 1:100 and assayed on an Illumina Eco using ABI SYBR Green PCR Master Mix for genes, GNAS and VIM, which were confirmed to be expressed stably based on initial data generated from the custom NanoString CodeSet (data not shown). The resulting Cq values for both genes were used to generate a dilution factor for each sample. Dilution factors were calculated by targeting each sample’s effective Cq value (post-dilution) to equal 16 for GNAS and 19 for VIM, which were the approximate values generated by the successful pilot samples (those with binding densities between 0.05 and 2.25 spots per square micron). Samples with Cq values greater than the target values were not diluted, and in samples where calculations resulted in significantly different dilution factors, the higher dilution factor was selected in order to avoid cartridge overloading. Following the dilution of the MTE PCR product, the DNA was denatured for 2 minutes at 94 °C then snap cooled on ice for 5 minutes prior to hybridization. The samples were hybridized overnight at 65 °C with the custom codeset described below.

### High-throughput measurement of lncRNA abundance

A custom nCounter™ Expression Assay codeset (Nanostring Technologies, Seattle, WA, USA) was used to quantify the abundance of 28 lncRNAs selected because of their role in the development or progression of pancreatic and other cancers after review of published literature^31–37^ *ADARB2*-*AS1*, *ANRIL*, *AS1DHRS4*, *BCYRN1*, *DDX6P*, *GAS5*, *GLIS3*-*AS1*, *H19*, *HOTAIR*, *HOTTIP*, *HOXD*-*AS1*, *HULC*, *LINC00244*, *LINC00469*, *LINC00472*, *LINC00491*, *lncRNA*-*p21*, *MALAT1*, *MEG3*, *PANDA*, *PPP3CB*, *PTENP1*, *PVT1*, *SRA*, *TERC*, *UCA1*, *XIST*, *αHIF*. The codeset also included positive controls, negative controls, three mRNA spike-in targets, four WBC cellular contamination targets (*APOE*, *CD68*, *CD2*, *and CD3*) three hemolysis/erythrocyte targets (*MB*, *NGB*, *CYGB*), and messenger RNA (mRNA) housekeeping genes (*ACTB*, *PGK1*, *and PPIB*). The NanoString cartridge was processed on the NanoString nCounter Prep Station using the high sensitivity protocol and scanned at the highest sensitivity setting (550 fields of view) on the nCounter Digital Analyzer. Data quality control was performed using the NanoString nSolver software, and were exported for normalization and analysis.

### Data processing and quality control

For each sample, background-corrected measures of lncRNA expression were estimated by subtracting the negative control average plus two standard deviation (SD) cut-point from the raw lncRNA counts. lncRNAs with less than 20% of samples above the negative control cut-point (ie. low-expression probes) were removed from downstream analysis. Possible WBC and erythrocyte sample contamination was evaluated using built-in controls. Based on manufacturer recommendations, data for each sample was normalized using the geometric mean of the three housekeeping genes (*ACTB*, *PGK1 and PPIB*), each of which appeared to be relatively invariant/stable in the dataset and to have less variability as compared to the mRNA spike-in genes. Normalized data was log2-transformed prior to signature selection.

### Statistical Analysis

Descriptive statistics were calculated using frequencies and percents for categorical variables and means and standard deviations (SD) for continuous variables. Data analysis was performed to identify a panel of lncRNAs that (a) differentiate between IPMN cases and non-diseased controls and (b) distinguish malignant (pathologically-confirmed as HG or invasive) from benign IPMNs (pathologically-confirmed as LG or MG).

### Identification of plasma lnRNA signatures

Linear models for microarray data (LIMMA)^74^ was used to identify lncRNAs that differentiate between IPMN cases and controls and between malignant and benign IPMNs, respectively. Since lncRNAs can be over- or under-expressed, we used principal component analysis (PCA) to combine the most deregulated lncRNAs and generate an overall ‘IPMN-risk score’ based on the first principal component (PC1), which accounts for the largest variability in the data and represents the overall combined effect of an IPMN-risk lncRNA signature. Specifically, IPMN-risk score, defined by PC1 as $\sum {w}_{i}{x}_{i}$, is a weighted average expression among the IPMN-risk lncRNAs, where *x*_*i*_ represents lncRNA *i* expression level, *w*_*i*_ is the corresponding weight (PC1’s loading coefficient for lncRNA *i*) with $\sum {w}_{i}^{2}=1$, and the *w*_*i*_ values maximize the variance of $\sum {w}_{i}{x}_{i}$. This approach has been used to derive gene signatures previously^22, 75–78^.

Receiver operating characteristic (ROC) curves were generated to measure the predictive power of the IPMN-risk signatures in discriminating between groups. Youdon method was used to determine the best threshold value for each model based on maximum of sum of sensitivities and specificities as the optimality criterion^79^. Estimates of sensitivity, specificity, positive predictive value (PPV), and negative predictive value (NPV) were calculated. For the analysis of malignant versus benign cases, we also conducted multivariable logistic regression analysis to assess whether the identified lncRNA signature was associated with malignant IPMN status independent of known prognostics factors (ie. main-duct involvement, lesion size, serum CA-19-9 level)^10^. Finally, to assess the extent to which data types in addition to plasma lncRNAs and standard clinical and radiologic features may augment correct prediction of malignant versus benign pathology, logistic regression models and ROC curves were also generated for a subset of cases with existing pre-operative plasma miRNA and radiomic data produced according to methods described in previous studies^22, 27^ and reviewed below. P values were adjusted with a false discovery rate (FDR) approach using the Benjamini and Hochberg method^80^. Additionally, to determine the ‘added value’ of the lncRNA, miRNA, and radiomic data, we calculated the integrated discrimination improvement (IDI) index, a magnitude of the reclassification probability improvement or worsening by a new test(s) over probability thresholds^81^.

### miRNA expression data

Preoperative plasma miRNA expression data was generated previously^22^ for 42 surgically-resected, pathologically-confirmed IPMN cases (21 malignant and 21 benign) using one 0.5-mL cryovial of plasma per case. Briefly, RNA spike-in miRNAs (synthetic control templates) were used and total RNA isolation was performed on 500 uL of plasma using the Plasma/Serum Circulating and Exosomal RNA Purification Mini Kit (Slurry Format) from Norgen Biotek (Ontario, Canada). The nCounter™ Human v2 miRNA Expression Assay Codeset (NanoString Technologies, Seattle, WA, USA) was used to quantify the abundance of a pre-defined panel of 800 human miRNAs and built-in controls, and raw miRNA counts underwent technical and biological normalization and log2-transformation. The most deregulated miRNAs were identified using the linear models for microarray data (LIMMA) method and a principal component analysis (PCA) approach (14). A focused analysis of the 42 IPMN cases showed that five miRNAs (miR-200a-3p, miR-1185-5p, miR-33a-5p, miR-574-3p, and miR-663b) had an AUC value of 0.73 (95% CI: 0.58–0.89) in discriminating between groups.

### CT acquisition and radiomic feature selection and extraction

CT and MRI-MRCP scans can be used to diagnose IPMNs, though MRI-MRCP is believed to be superior for optimal management^82^. In our series of surgically-resected, pathologically-confirmed IPMN cases, more cases had available pre-operative CT images versus pre-operative MRI-MRCP images. Specifically, preoperative CT images were available for 38 of the 42 pathologically-confirmed IPMN cases who also had available matched preoperative miRNA expression data generated previously^22^. CT images were obtained from Moffitt’s GE Centricity Picture Archiving and Communication System (PACS). CT images were reviewed for standard radiologic features encompassing ‘high-risk stigmata’ and ‘worrisome features’ represented in consensus guidelines^10^. Axial venous phase images (3 mm) were used for most patients the region of interest (ROI) by helping to outline the peripheral margin of tumors in their entirety, capturing both solid (nodular) and cystic components. The radiomics team then marked the ROI using Definiens/GE AWS Advanced Visualization software. The entire tumors were identified (solid and cystic components) using a semi-manual version of a single click semi-automated ensemble segmentation algorithm within the Definiens Developer XD (Munich, Germany) software platform. Target lesions were segmented, with a second radiologist finalizing the segmentation boundaries on the CT slices. We then extracted categories of 18 non-texture and 94 texture features. Non-texture features measure tumor size (volume, diameter, border length), shape (compactness, asymmetry), and location, whereas texture features measure properties such as smoothness, coarseness, and regularity. We focused on evaluating two-dimensional (2D) quantitative features in the middle CT slice. In house algorithms for feature extraction and quantification of segmented regions were implemented by custom routines in the Definiens Platform. Logistic regression, PCA, and cross-validation analyses were used to examine associations between features and IPMN pathology. Fourteen features, most of which were textural, differentiated malignant from benign IPMNs and collectively had an AUC value of 0.77.

### Cross-validation analysis

To evaluate model performance, repeated (10,000 times) 10-fold cross validation was performed. The average and 95% confidence intervals of accuracy, sensitivity, specificity, PPV and NPV were estimated. In each 10-fold cross-validation, data were split into 10 subsets. By holding one subset of data (test set), the remaining 9 subsets were used as a training set to build a model for prediction evaluation in the test set. The process continued until each subset was used as the test set. By testing the model on a test set (not used in estimation), cross-validation aimed to reduce over-fitting. All statistical analyses were performed using SAS version 9.4 and R version 3.2.5.

## Electronic supplementary material

Supplementary Information
