# Supplementary material for: Linc-ing Circulating Long Non-coding RNAs to the Diagnosis and Malignant Prediction of Intraductal Papillary Mucinous Neoplasms of the Pancreas
Source: Sci Rep. 2017 Sep 5;7:10484. doi: 10.1038/s41598-017-09754-5 (PMC5585319; doi:10.1038/s41598-017-09754-5)
Supplement: Supplementary file 1 — Supplementary Information [file 41598_2017_9754_MOESM1_ESM.pdf]

Supplementary Information

Linc-ing Circulating Long Non-coding RNAs to the Diagnosis and Malignant Prediction of Intraductal Papillary Mucinous Neoplasms of the Pancreas

Short Title: Plasma lncRNAs and Intraductal Papillary Mucinous Neoplasms

Jennifer B. Permuth[1,2], Dung-Tsa Chen[3], Sean J. Yoder[4], Jianong Li[3], Andrew T. Smith[4], Jung W. Choi[5], Jongphil Kim[3], Yoganand Balagurunathan[6], Kun Jiang[7], Domenico Coppola[7], Barbara A. Centeno[7], Jason Klapman[2], Pam Hodul[2], Florian Karreth[8], Jose Trevino[9], Nipun Merchant[10], Anthony Magliocco[7], Mokenge P. Malafa[2] , and Robert Gillies[6]

Departments of [1]Cancer Epidemiology, [2]Gastrointestinal Oncology, [3]Biostatistics and Bioinformatics,[4]Molecular Genomics Core Facility, [5]Diagnostic Imaging and Interventional Radiology, [6]Cancer Imaging and Metabolism, [7]Anatomic Pathology, [8]Molecular Oncology, Moffitt Cancer Center and Research Institute, Tampa, Florida
[9]Department of Surgery, Division of General Surgery, University of Florida Health Sciences Center, Gainesville, Florida
[10]Department of Surgery, Sylvester Comprehensive Cancer Center at the University of Miami Miller School of Medicine, Miami, Florida

Supplementary Figure S1

Supplementary Table S1

Supplementary Table S2

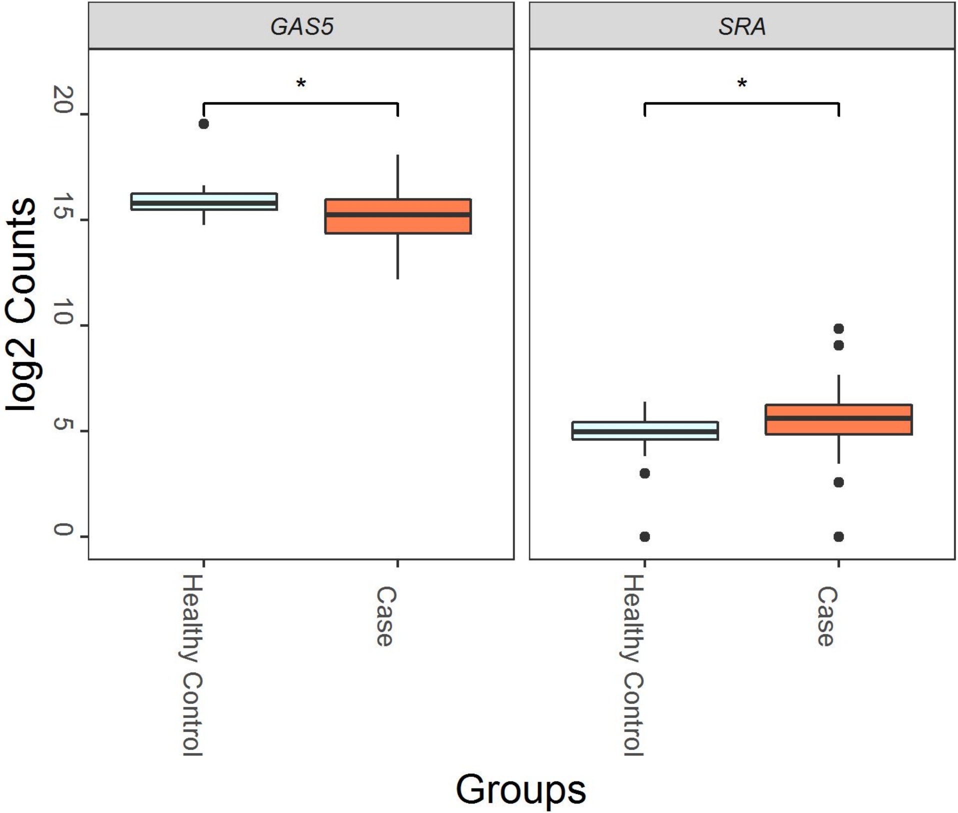

GAS5
SRA
*
*
log2 Counts
20
15
10
5
0
Healthy Control
Case
Healthy Control
Case
Groups

Supplementary Table S1. LncRNA expression in IPMN cases versus controls, sorted by the most statistically significant difference between groups

LncRNA	Overall mean	Control mean (n=22)	Case mean (n=51)	Fold Change Cases versus Controls	p value Control	FDR
GAS5	15.4	15.9	15.2	1.0	**0.026**	0.632
SRA	5.3	4.6	5.5	1.2	**0.045**	0.632
GLIS3-AS1	12.0	12.6	11.7	0.9	0.111	0.836
AS1DHRS4	13.5	13.9	13.4	1.0	0.119	0.836
PTENP1	6.1	5.3	6.5	1.2	0.175	0.859
HOTTIP	5.6	5.1	5.8	1.1	0.243	0.859
PPP3CB	13.3	12.8	13.5	1.1	0.269	0.859
UCA1	12.2	12.6	12.1	1.0	0.277	0.859
ADARB2-AS1	10.5	10.8	10.4	1.0	0.301	0.859
aHIF	9.4	9.1	9.6	1.1	0.307	0.859
DDX6P	2.7	2.2	2.9	1.3	0.406	0.949
MEG3	10.4	10.8	10.3	1.0	0.407	0.949
LINC00244	0.7	0.4	0.9	2.0	0.482	1.000
lncRNA-p21	5.7	5.4	5.9	1.1	0.512	1.000
H19	15.0	15.1	15.0	1.0	0.656	1.000
MALAT1	10.7	10.5	10.7	1.0	0.692	1.000
XIST	0.9	0.8	0.9	1.1	0.759	1.000
LINC00469	9.7	9.8	9.6	1.0	0.768	1.000
HOXD-AS1	7.1	7.0	7.1	1.0	0.782	1.000
PANDA	8.5	8.7	8.4	1.0	0.787	1.000
LINC00491	9.8	9.6	9.9	1.0	0.862	1.000
TERC	9.2	9.3	9.2	1.0	0.881	1.000
LINC00472	10.4	10.5	10.4	1.0	0.909	1.000
BCYRN1	0.2	0.3	0.2	0.7	0.916	1.000
PVT1	12.2	12.4	12.2	1.0	0.919	1.000
ANRIL	11.8	12.0	11.7	1.0	0.942	1.000
HULC	2.2	2.5	2.1	0.9	0.978	1.000
HOTAIR	6.5	6.4	6.5	1.0	1.000	1.000

Supplementary Table S2. Diagnostic permormance of preliminary models to predict malignant IPMN pathology in the study cohort based on 10-fold cross validation

Variables	Accuracy	Sensitivity	Specificity	PPV	NPV
Gender	0.49 (0.39-0.55)	0.07 (0-0.21)	0.83 (0.65-1)	0.21 (0-0.5)	0.52 (0.46-0.56)
Jaundice	0.58 (0.52-0.65)	0.14 (0-0.29)	0.94 (0.94-1)	0.61 (0-0.8)	0.57 (0.53-0.62)
High risk stigmata (HRS)	0.84 (0.84-0.84)	0.86 (0.86-0.86)	0.82 (0.82-0.82)	0.8 (0.8-0.8)	0.87 (0.87-0.87)
Worrisome features (WF)	0.52 (0.45-0.58)	0.16 (0-0.43)	0.81 (0.53-1)	0.41 (0.33-0.6)	0.54 (0.5-0.57)
8-lncRNA signature	0.72 (0.65-0.77)	0.65 (0.5-0.71)	0.77 (0.71-0.82)	0.7 (0.62-0.77)	0.73 (0.67-0.78)
5-miRNA signature	0.65 (0.55-0.74)	0.67 (0.43-0.86)	0.64 (0.53-0.76)	0.6 (0.5-0.69)	0.71 (0.58-0.83)
14-feature radiomic signature	0.7 (0.61-0.77)	0.66 (0.5-0.79)	0.73 (0.71-0.76)	0.67 (0.58-0.73)	0.72 (0.63-0.81)
lncRNA, miRNA	0.76 (0.68-0.81)	0.74 (0.64-0.79)	0.77 (0.71-0.82)	0.73 (0.65-0.79)	0.78 (0.71-0.82)
lncRNA, miRNA,radiomic	0.66 (0.61-0.71)	0.68 (0.57-0.79)	0.65 (0.65-0.65)	0.61 (0.57-0.65)	0.72 (0.65-0.79)
HRS,WF,lncRNA	0.84 (0.84-0.84)	0.86 (0.86-0.86)	0.82 (0.82-0.82)	0.8 (0.8-0.8)	0.87 (0.87-0.87)
HRS,WF,miRNA	0.81 (0.81-0.84)	0.79 (0.79-0.79)	0.83 (0.82-0.88)	0.79 (0.79-0.85)	0.82 (0.82-0.83)
HRS,WF,Radiomic	0.84 (0.84-0.84)	0.86 (0.86-0.86)	0.82 (0.82-0.82)	0.8 (0.8-0.8)	0.87 (0.87-0.87)
HRS,WF,lncRNA,miRNA	0.78 (0.74-0.84)	0.78 (0.71-0.79)	0.78 (0.76-0.88)	0.75 (0.69-0.85)	0.81 (0.76-0.83)
HRS,WF,lncRNA,Radiomic	0.84 (0.84-0.84)	0.86 (0.86-0.86)	0.82 (0.82-0.82)	0.8 (0.8-0.8)	0.87 (0.87-0.87)
HRS,WF,lncRNA,miRNA,Radiomic	0.78 (0.71-0.84)	0.77 (0.64-0.79)	0.78 (0.71-0.88)	0.74 (0.69-0.83)	0.81 (0.72-0.83)
HRS,WF,gender, jaundice,lncRNA,Radiomic,miRNA	0.75 (0.68-0.81)	0.73 (0.57-0.86)	0.76 (0.71-0.82)	0.71 (0.64-0.79)	0.78 (0.68-0.87)
WF,lncRNA	0.67 (0.55-0.74)	0.56 (0.36-0.71)	0.76 (0.65-0.82)	0.66 (0.5-0.75)	0.68 (0.58-0.76)
WF,Radiomic	0.62 (0.52-0.68)	0.51 (0.36-0.64)	0.7 (0.59-0.76)	0.59 (0.45-0.67)	0.64 (0.55-0.71)
WF,miRNA	0.66 (0.55-0.74)	0.55 (0.36-0.64)	0.75 (0.65-0.82)	0.65 (0.5-0.75)	0.67 (0.57-0.74)
WF,lncRNA,Radiomic	0.64 (0.55-0.68)	0.63 (0.5-0.71)	0.64 (0.59-0.71)	0.59 (0.5-0.64)	0.68 (0.59-0.73)
WF,lncRNA,miRNA	0.66 (0.58-0.74)	0.58 (0.43-0.71)	0.74 (0.65-0.82)	0.64 (0.54-0.75)	0.68 (0.61-0.75)
WF,lncRNA,miRNA,Radiomic	0.77 (0.68-0.84)	0.71 (0.64-0.79)	0.82 (0.71-0.88)	0.76 (0.67-0.85)	0.77 (0.71-0.83)
WF,gender,Jaundice,lncRNA,Radiomic,miRNA	0.78 (0.68-0.87)	0.69 (0.57-0.79)	0.86 (0.71-0.94)	0.8 (0.67-0.92)	0.77 (0.68-0.84)

Abbreviations: AUC=Area underneath the curve; PPV=positive predictive value; NPV=negative predictive value
